# Clinical Predictors of Aspirin Resistance in Patients with Type 2 Diabetes: A Systematic Review and Meta-Analysis

**DOI:** 10.31083/RCM26009

**Published:** 2025-01-20

**Authors:** Fan Zhang, Hongyan Zheng

**Affiliations:** ^1^Department of Cardiology, Nanjing Drum Tower Hospital, The Affiliated Hospital of Nanjing University Medical School, 210008 Nanjing, Jiangsu, China

**Keywords:** aspirin resistance, acetylsalicylic acid, diabetes mellitus, risk factors, meta-analysis

## Abstract

**Background:**

Aspirin treatment is recommended as a secondary prevention strategy and could be a potential primary prevention strategy for cardiovascular disease (CVD) in patients with type 2 diabetes mellitus (T2DM). However, aspirin resistance is notably common among diabetic patients, compromising the efficacy of aspirin treatment. Hence, our study sought to assess the clinical predictors of aspirin resistance (AR) in T2DM patients.

**Methods:**

We conducted a systematic search of three major medical databases (PubMed, Embase, and Cochrane Library) to identify relevant articles up to September 17, 2024. Details of publications and investigated parameters were extracted from the selected studies. The meta package in the R language software was utilized to synthesize the evidence concerning clinical predictors of AR. We applied either a fixed- or random effects model based on the heterogeneity observed among the included studies. The pooled results were visually displayed using forest plots.

**Results:**

In total, 10 publications were finally included in our study (n = 2113 patients). AR was predominantly linked to specific laboratory parameters, particularly those indicative of heightened insulin resistance and inadequate lipid management. Specifically, the laboratory parameters associated with AR included fasting glucose level (mean difference (MD) = 8.21; 95% confidence interval (CI) = 2.55 to 13.88), glycated hemoglobin (MD = 0.22; 95% CI = 0.06 to 0.38), high-density lipoprotein (HDL) level (MD = –2.02; 95% CI = –3.62 to –0.42), low-density lipoprotein (LDL) level (MD = 7.00; 95% CI = 2.87 to 11.13), total cholesterol level (MD = 9.52; 95% CI = 4.37 to 14.67), and triglyceride levels (MD = 12.51; 95% CI = 3.47 to 21.55).

**Conclusions:**

Markers associated with dyslipidemia and blood glucose levels are robust indicators of AR in individuals with T2DM. These findings imply that assessing lipid and glucose regulation could enhance the development of personalized preventive approaches for vascular complications linked to diabetes.

**The PROSPERO registration:**

CRD42023388170, https://www.crd.york.ac.uk/PROSPERO/display_record.php?RecordID=388170

## 1. Introduction

Type 2 diabetes mellitus (T2DM) is recognized as a significant and independent risk factor for coronary heart disease [1, 2]. Vascular events remain the primary cause of both mortality and morbidity in individuals with T2DM [3]. According to the guidelines issued by the American Diabetes Association (ADA) [4], acetylsalicylic acid (ASA) is recommended as a secondary prevention measure for T2DM patients with a prior history of atherosclerotic cardiovascular disease (CVD). ASA may also be contemplated as a primary prevention strategy for diabetic individuals at heightened cardiovascular risk [4]. Despite the recommended use of aspirin therapy in patients with T2DM, the efficacy of aspirin therapy in primary CVD prevention is currently suboptimal [5]. Specifically, as a secondary prevention strategy, the reduction in cardiovascular event risk in T2DM patients following ASA was less than 10%, which contrasts with a greater than 20% decrease observed in non-diabetic patients [6]. The diminished efficacy of aspirin use as an antiplatelet agent is attributed to aspirin resistance (AR), characterized by high platelet reactivity (HPR) and poorly inhibited thromboxane synthesis *in vivo* despite administering the recommended dose of aspirin. Some studies showed that AR occurred more frequently in patients with diabetes [7, 8]. The clinical implications of this inadequate platelet suppression could be significant, as AR has been associated with an elevated risk of adverse cardiovascular events in individuals with a prior history of myocardial infarction, as well as a more than threefold increase in the risk of adverse primary outcomes in patients with chronic coronary syndromes [9, 10].

An essential mechanism through which aspirin exerts its antiplatelet effect is by inhibiting the cyclooxygenase-1-related (COX-1) pathway [11]. In T2DM patients, prolonged hyperglycemia, resulting from insulin resistance and metabolic disorders, mediates the accumulation of oxidative stress and triggers the damage of endothelium by creating an imbalance between vasodilators and vasoconstrictors [12]. Endothelial dysfunction and an associated chronic low-grade inflammation state accelerate the generation of proinflammatory cytokines, acute phase proteins, adipokines, and chemokines [13]. Accordingly, there is an increase in platelet turnover and a higher count of young, reticulated platelets [13, 14]. Although unacetylated COX-1 and COX-2 from newly-formed platelets are believed to play a pivotal role in AR, a broadly applicable and widely recognized comprehensive mechanism remains elusive. The complicated interactions between platelet activation and the pathogenic events occurring in patients with high platelet reactivity make it challenging to interpret current mechanistic information as clinically formative indicators [15]. If statistically verified by available evidence, such clinical predictors of AR could assist in providing personalized therapies, leading to more favorable clinical outcomes. However, to our knowledge, no available clinical trial study is currently attempting to determine clinical predictors of AR in patients with T2DM. Some observational studies suggested that demographic characteristics, such as age and body mass index (BMI), are potential predictive factors [16, 17]. Conversely, other studies have proposed diverse laboratory parameters, including glycemic levels and serum lipid profiles, as possible markers of AR in T2DM patients [18, 19]. A study of diabetic patients taking ASA daily found that AR is linked to lipid dysfunction and a history of smoking [20]. However, current findings are inconsistent and mainly based on observed evidence. Moreover, there is a shortage of comprehensive reports synthesizing evidence of clinical predictors of AR within patients with T2DM.

Therefore, this study aimed to review the current literature on clinical predictors of AR in diabetic patients, to inform decision-making regarding suitable interventions for preventing diabetes-related complications, and to improve the likelihood of more favorable clinical outcomes.

## 2. Materials and Methods

This systematic review and meta-analysis was conducted according to the Preferred Reporting Items for Systematic Reviews and Meta-Analyses (PRISMA) 2020 guidelines [21]. The review protocol was registered in PROSPERO (ID: CRD42023388170).

### 2.1 Eligibility Criteria

The inclusion criteria comprised (1) a confirmed diagnosis of T2DM; (2) administration of aspirin at a minimum daily dose of 75 mg; (3) identification of ASA responders by analyzing platelet aggregation among aspirin consumers; (4) comparison of demographics data and primary laboratory data between ASA responders and ASA non-responders among diabetic patients. The exclusion criteria included (1) use of antiplatelet medications other than aspirin; (2) use of anticoagulants; (3) chronic use of non-steroidal anti-inflammatory drugs (NSAIDs); (4) presence of coagulation disorders; (5) non-English publications.

### 2.2 Information Source and Search Strategy

Two independent reviewers conducted a comprehensive search of three major databases (PubMed, Embase, and Cochrane Library) up to September 17, 2024, to identify relevant articles investigating the correlation between aspirin resistance and clinical characteristics in patients diagnosed with T2DM. The search utilized Medical Subject Headings (MeSH) terms and keywords such as “aspirin”, “acetylsalicylic acid”, “platelet reactivity”, “antiplatelet”, “platelet hyperactivation”, “aspirin resistance”, “diabetes mellitus”, “diabetes”, “diabetic”, and “T2DM”. The detailed search strategy can be found in Table 1.

**Table 1.** **Search strategies**.

Database	Search terms	Records identified
PubMed	(((((((((‘platelet reactivity’) OR (‘platelet activation’)) OR (‘antiplatelet activity’)) OR (‘platelet hyperactivation’)) OR (‘platelet hyperactivity’)) OR (‘aspirin resistance’)) OR (‘resistance to aspirin’)) OR (‘acetylsalicylic acid resistance’)) OR (‘resistance to acetylsalicylic acid’)) AND (((((‘diabetes mellitus’[MeSH Terms])) OR (‘diabetes’)) OR (‘diabetic’)) OR (‘T2DM’)) AND ((((((‘Aspirin’[MeSH Terms])) OR (‘acetylsalicylic acid’ [MeSH Terms])) OR (‘salicylate’)) OR (‘aspirin’)) OR (‘acetylsalicylic acid’))	1029
Cochrane Library	#1 MeSH descriptor: [platelet activation] explode all trees	132
	#2 ‘platelet reactivity’	
	#3 ‘antiplatelet activity’	
	#4 ‘platelet hyperactivation’	
	#5 ‘platelet hyperactivity’	
	#6 ‘aspirin resistance’	
	#7 ‘resistance to aspirin’	
	#8 ‘acetylsalicylic acid resistance’	
	#9 ‘resistance to acetylsalicylic acid’	
	#10 #1 OR #2 OR #3 OR #4 OR #5 OR #6 OR #7 OR #8 OR #9	
	#11 MeSH descriptor: [diabetes mellitus] explode all trees	
	#12 ‘diabetic’	
	#13 ‘T2DM’	
	#14 #11 OR #12 OR #13	
	#15 MeSH descriptor: [aspirin] explode all trees	
	#16 ‘acetylsalicylic acid’	
	#17 ‘salicylate’	
	#18 #15 OR #16 OR #17	
	#19 #10 AND #14 AND #18	
Embase	(‘thrombocyte activation’/exp OR ‘platelet reactivity’ OR ‘antiplatelet activity’ OR ‘platelet hyperactivation’ OR ‘platelet hyperactivity’ OR ‘aspirin resistance’ OR ‘resistance to aspirin’ OR ‘acetylsalicylic acid resistance’ OR ‘resistance to acetylsalicylic acid’) AND (‘diabetes mellitus’/exp OR ‘diabetic’ OR ‘T2DM’) AND (‘aspirin’ OR ‘acetylsalicylic acid’/exp OR ‘salicylate’)	1490

Abbreviations: MeSH, Medical Subject Headings; T2DM, type 2 diabetes mellitus.

### 2.3 Selection Process

Two reviewers independently screened the titles and abstracts of studies identified in the initial search. After removing duplicates, articles were categorized as ineligible, potentially eligible, or eligible based on the specified inclusion criteria. The two reviewers subsequently retrieved and assessed the full texts of potential articles for eligibility. Any discrepancies between the reviewers were resolved through group discussion. The selection process was facilitated using EndNote X9 software (Clarivate Plc., London, United Kingdom).

### 2.4 Data Collection Process

Two authors independently conducted data extraction. Any discrepancies regarding the potential extraction of data items were resolved through group discussions until a consensus was reached. The extracted summary data encompassed publication details (title, study type, authors, publication year, and journal), study designs, inclusion and exclusion criteria, sample characteristics, aspirin dosing regimens, diabetes treatment, coexisting conditions, treatment of concomitant diseases, methods for defining and detecting platelet reactivity, prevalence of aspirin resistance, laboratory findings, and associations reported between patient characteristics and heightened platelet activity. Clinical features included in the meta-analysis were those investigated in at least three studies.

### 2.5 Study Risk of Bias Assessment

Given that all the studies were observational, two reviewers independently assessed the risk of bias using the Newcastle–Ottawa scale (NOS) score [22] to determine the quality of observational studies. Cross-sectional studies scoring ≥7, 6, and ≤5 were considered high, intermediate, and low quality, respectively.

### 2.6 Effect Measures

The main outcome of interest in our meta-analysis was the correlation between AR and clinical features in patients with T2DM. Effect measures included risk ratios, mean differences, and their corresponding 95% confidence intervals (CIs) for the aspirin responder and non-responder groups.

### 2.7 Statistical Analysis

R version 4.2.1 (R Foundation for Statistical Computing, Vienna, Austria) was used to analyze the data, and R package meta version 7.0 (Guido Schwarzer, Freiburg, BW, Germany) was applied to integrate the data [23]. Continuous variables are presented as the mean ± standard deviation, while categorical variables are expressed as numbers and percentages. In cases where studies reported continuous variables as median and interquartile ranges (IQRs), the method described by Wan *et al*. [24] was utilized to estimate the mean and standard deviations. When patients were stratified into multiple groups based on platelet reactivity (e.g., high, medium, and low), these groups were consolidated into two categories: the aspirin-resistance positive (AR+) group (patients with high platelet reactivity) and aspirin-resistance negative (AR–) group (patients with medium or low platelet reactivity). Subsequently, the mean and standard deviations for these two merged groups were calculated. I^2^ statistics and Cochran’s Q test were applied to assess the heterogeneity of the pooled results. Heterogeneity was deemed substantial for I^2^ values ≥50% and *p*-values ≤ 0.1, necessitating the utilization of the random effects model. In cases where these criteria were not met, a meta-analysis was conducted employing a fixed effects model. The combined results were presented as forest plots. The Galbraith plot was then used to examine the potential outliers and heterogeneity among studies. Subgroup analysis was conducted to explore the discrepancy between subgroups. Meta-regression analysis was used to investigate the source of heterogeneity. Given the potentially substantial impact of varied AR detection methods on the outcomes, sensitivity analysis was performed by systematically excluding each AR detection method to evaluate the influence on heterogeneity. It should be noted that a subgroup analysis using AR detection methods was not applicable. Since there were six detection methods from 10 studies, many subgroups involved only one study, making data synthesis invalid. In addition, reporting bias was assessed through Egger’s test and funnel plots [25]. A *p*-value < 0.05 was considered statistically significant.

### 2.8 Certainty Assessment

The Grading of Recommendations, Assessment, Development, and Evaluations (GRADE) system was used to assess the quality of evidence in our study [26]. Given that the included studies were observational, the initial level of certainty for all ratings was considered low. Evidence ratings were subject to potential upgrading or downgrading based on specific characteristics of the analyzed studies, resulting in final grades of very low, low, moderate, or high certainty.

## 3. Results

### 3.1 Study Selection

**Fig. 1.**
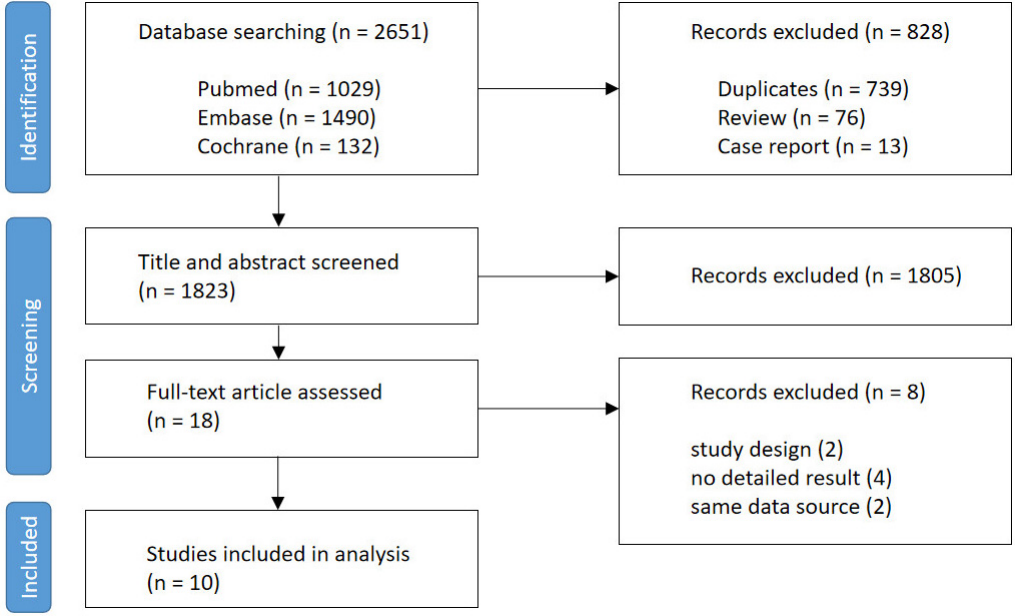
**Flow diagram of the study selection process**.

A flow diagram explaining the study selection is presented in Fig. 1. Initially, 2651 studies were identified through the database search. Following the removal of 739 duplicate studies, 76 reviews, and 13 case reports, the titles and abstracts of 1823 articles were screened based on the predefined inclusion and exclusion criteria, resulting in 18 papers selected for a full-text review. Among these, two studies were excluded for focusing on comparing platelet reactivity measurement methods, four were eliminated for lacking detailed data, and two were excluded for sharing the same data source with other articles. Eventually, 10 eligible articles were included in this meta-analysis.

### 3.2 Study Characteristics and Quality Evaluation

The included articles and their main features are summarized in Table 2 (Ref. [16, 17, 18, 19, 20, 27, 28, 29, 30, 31]). All studies were cross-sectional investigations involving 2113 patients diagnosed with T2DM and receiving aspirin treatment. Sample sizes across the studies ranged from 48 to 1045 individuals. Among the 2113 patients, 380 were classified in the AR+ group, while 1733 were categorized in the AR– group. The prevalence of AR in the included studies varied between 10% and 47%. The mean ages of the patients ranged from 60.5 to 67.7 years, with the proportion of female patients ranging from 31% to 59%. The primary method for assessing high platelet activation (HPA) was platelet function analyzer (PFA)-100 closure time, with additional measures such as light transmission aggregometry (LTA), thromboelastography (TEG), Multiplate analyzer (MPA), VerifyNow system (VNS), and serum thromboxane B2 (TXB2) immunoassay (STI) also utilized in some studies. The clinical characteristics analyzed in this meta-analysis were categorized into four main groups (Table 3 (Ref. [16, 17, 18, 19, 20, 27, 28, 29, 30, 31])): demographic characteristics, concurrent medication, coexisting conditions, and laboratory results. Laboratory results were further organized into three subcategories, encompassing diabetic parameters, lipid control parameters, and other laboratory parameters.

**Table 2.** **Characteristics of included studies**.

Author, year	Country	Sample size/AR group (%)	Age (years)	Female (%)	AR detection method	Aspirin dose
Barale *et al*. [17], 2020	Italy	103/24 (23)	64.5 ± 7	50 (49)	PFA-100	100 mg/day
Paven *et al*. [27], 2020	France	116/27 (23)	65 ± 9	36 (31)	LTA	75 mg/day
Habizal *et al*. [28], 2015	Malaysia	69/12 (17)	61 ± 7.6	25 (36)	thromboelastography	75–150 mg/day
Tasdemir *et al*. [19], 2014	Turkey	93/39 (42)	60.5 ± 11.9	55 (59)	PFA-100	100 mg/day
Łabuz-Roszak *et al*. [20], 2014	Poland	96/45 (47)	65.3 ± 8.1	48 (50)	Multiplate analyzer	75–150 mg/day
Kim *et al*. [29], 2014	Korea	1045/105 (10)	61.4 ± 9.4	443 (42)	VerifyNow system	100 mg/day
Kaplon-Cieslicka *et al*. [16], 2014	Poland	186/45 (24)	67.7 ± 8.7	94 (51)	Serum TXB_2_ immunoassay	75 mg/day
Postula *et al*. [30], 2012	Poland	185/35 (19)	66.4 ± 8.7	100 (54)	VerifyNow system	75 mg/day
Cohen *et al*. [18], 2008	USA	48/11 (23)	67 ± 16	28 (58)	PFA-100	81–325 mg/day
Fateh-Moghadam *et al*. [31], 2005	Germany	172/37 (22)	62.3 ± 8.9	65 (38)	PFA-100	100 mg/day

Abbreviations: AR, aspirin resistance; PFA, platelet function analyzer; LTA, light transmission aggregometry; TXB2, thromboxane B2.

**Table 3.** **Parameters examined in this meta-analysis**.

Examined parameters			Barale *et al*. [17], 2020	Paven *et al*. [27], 2020	Habizal *et al*. [28], 2015	Tasdemir *et al*. [19], 2014	Łabuz-Roszak *et al*. [20], 2014	Kim *et al*. [29], 2014	Kaplon-Cieslicka *et al*. [16], 2014	Postula *et al*. [30], 2012	Cohen *et al*. [18], 2008	Fateh-Moghadam *et al*. [31], 2005	Total
1. Demographic characteristics													
	1.1. Age (years)		√	√	√	√	√	√	√	√	√	√	10
	1.2. Female gender, n (%)		√	√	√	√	√	√	√	√	√	√	10
	1.3. BMI (kg/m^2^)		√	√		√	√	√	√	√	√	√	9
	1.4. Current smoker, n (%)			√	√	√	√	√	√	√	√	√	9
2. Concurrent medications													
	2.1. ACE inhibitors, n (%)			√		√	√		√	√			5
	2.2. Beta-blockers, n (%)			√		√	√		√	√			5
	2.3. Calcium channel blockers, n (%)						√		√	√			3
	2.4. Statins, n (%)			√		√	√		√	√			5
3. Coexisting conditions													
	3.1. Coronary heart disease, n (%)			√		√	√	√	√	√	√		7
	3.2. Hypertension, n (%)			√	√		√		√	√		√	6
	3.3. Previous MI, n (%)			√			√		√	√			4
	3.4. Previous stroke, n (%)			√	√		√	√	√	√			6
4. Laboratory results													
	4.1. Diabetic parameters												
		4.1.1. Fasting glucose (mg/dL)	√	√	√	√	√	√	√	√			8
		4.1.2. HbA1c (%)	√	√	√	√	√	√	√	√	√	√	10
		4.1.3. HOMA-IR		√				√	√				3
		4.1.4. Insulin (µIU/mL)		√				√	√				3
	4.2. Lipid control parameters												
		4.2.1. HDL (mg/dL)	√	√	√		√	√	√	√			7
		4.2.2. LDL (mg/dL)	√		√		√	√	√	√			6
		4.2.3. TC (mg/dL)	√			√	√	√	√	√	√		7
		4.2.4. TGs (mg/dL)	√	√	√		√	√	√	√			7
	4.3. Other laboratory parameters												
		4.3.1. Creatinine (µmol/L)		√		√	√	√		√			5
		4.3.2. eGFR (mL/min/1.73 m^2^)		√				√	√	√			4
		4.3.3. Hemoglobin (g/dL)		√			√	√	√	√			5
		4.3.4. MPV (fL)	√	√		√			√	√			5
		4.3.5. PLT (1000/mm^3^)	√	√		√	√		√	√			6

Abbreviations: BMI, body mass index; ACE, angiotensin-converting enzyme; MI, myocardial infarction; HbA1c, glycated hemoglobin; HOMA-IR, Homeostasis Model Assessment-Insulin Resistance; HDL, high-density lipoprotein; LDL, low-density lipoprotein; TC, total cholesterol; TGs, triglycerides; eGFR, estimated glomerular filtration rate; MPV, mean platelet volume; PLT, platelet count.

The risk of bias was assessed using the NOS score and scoring criteria, with the NOS scores presented in **Supplementary Table 1**. The average NOS score of the 10 articles was 6.4. Among these, four studies [16, 17, 18, 31] achieved a NOS score of 7 or higher, indicating a low risk of bias. The remaining six studies [19, 20, 27, 28, 29, 30] obtained a NOS score of 6, suggesting a moderate risk of bias. Notably, all articles had a NOS score of 6 or above. Regarding the GRADE rating, all included reports had a low certainty of evidence due to the nature of observational studies. The funnel plot (**Supplementary Fig. 1**) and Egger’s test (**Supplementary Table 2**) demonstrated no significant differences in any of the comparisons (*p* > 0.05), suggesting a low probability of publication bias. In conclusion, the overall quality of the included studies was acceptable and consistent, indicating a low risk of being the source of heterogeneity.

### 3.3 Results of Meta-Analysis

#### 3.3.1 Correlation between AR and Demographic Characteristics

**Fig. 2.**
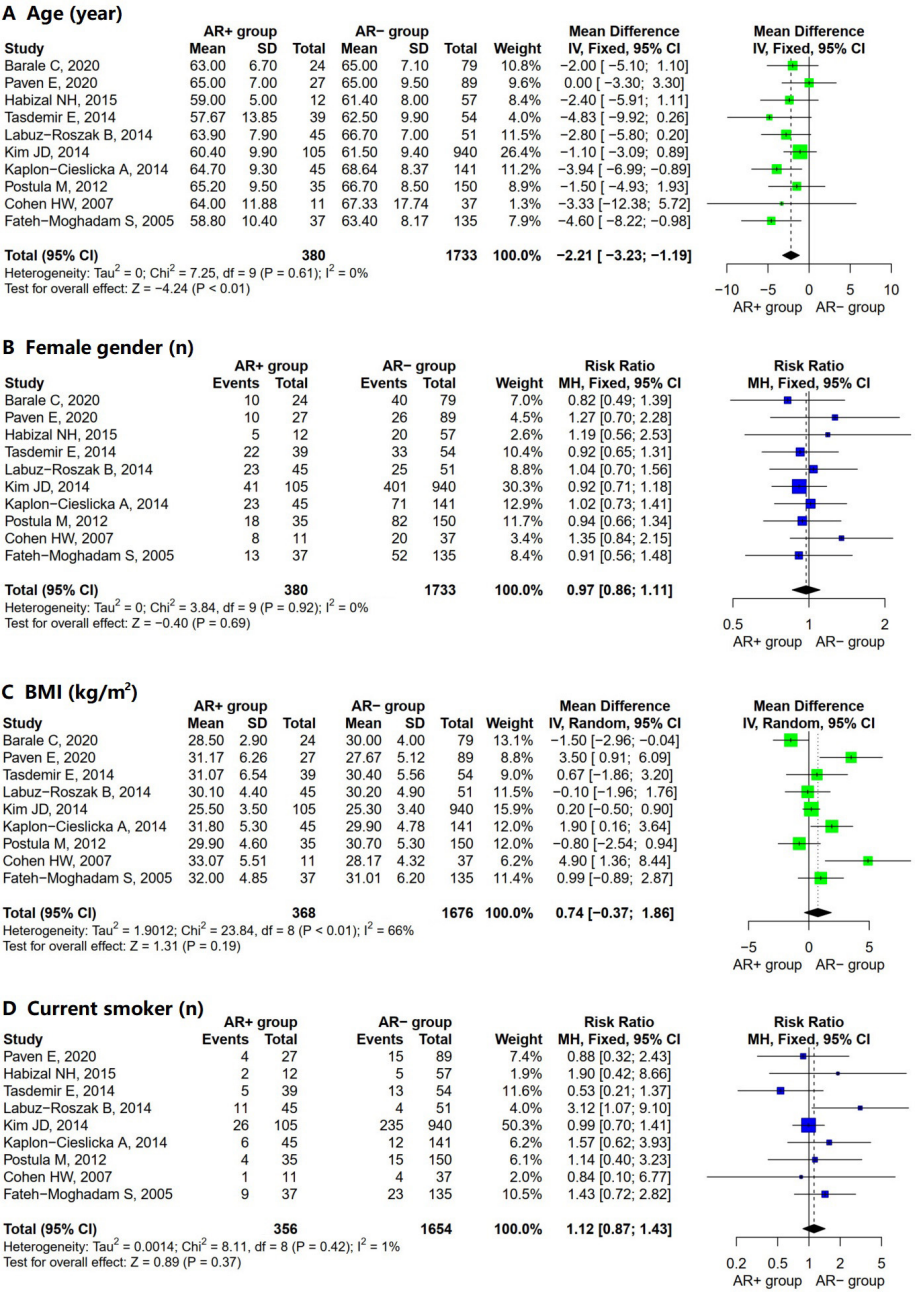
**Forest plot for AR+ vs. AR– regarding (A) age, (B) female gender, (C) BMI, and (D) current smoker**. Abbreviations: SD, standard deviation; MH, Mantel–Haenszel model; IV, inverse variance method; CI, confidence interval; AR, aspirin resistance; BMI, body mass index.

Most of the included studies extensively detailed the evaluation of demographic characteristics (Table 3). The pooled results showed that the AR+ group was younger than the AR– group (Fig. 2A; MD = –2.21; 95% CI = –3.23 to –1.19; I^2^ = 0%; *p* = 0.61). However, the meta-analysis did not reveal significant differences between the two groups concerning other demographic factors, including female gender (Fig. 2B; OR = 0.97; 95% CI = 0.86 to 1.11; I^2^ = 0%; *p* = 0.92), BMI (Fig. 2C; MD = 0.74; 95% CI = –0.37 to 1.86; I^2^ = 66%; *p* < 0.01), and current smoker (Fig. 2D; OR = 1.12; 95% CI = 0.87 to 1.43; I^2^ = 1%; *p* = 0.42).

#### 3.3.2 Correlation between AR and Concurrent Medications

Concurrent medications were reported in about half of the 10 studies included in the analysis (Table 3). Our data analysis did not reveal any significant differences in terms of concurrent medications between the AR– group and the AR+ group. The concurrent medications considered were angiotensin-converting enzyme (ACE) inhibitors (**Supplementary Fig. 2A**; OR = 1.02; 95% CI = 0.86 to 1.21; I^2^ = 50%; *p* = 0.09), beta-blockers (**Supplementary Fig. 2B**; OR = 1.07; 95% CI = 0.93 to 1.23; I^2^ = 0%; *p* = 0.72), calcium antagonists (**Supplementary Fig. 2C**; OR = 1.18; 95% CI = 0.73 to 1.91; I^2^ = 64%; *p* = 0.06), and statins (**Supplementary Fig. 2D**; OR = 0.88; 95% CI = 0.78 to 1.00; I^2^ = 0%; *p* = 0.56).

#### 3.3.3 Correlation between AR and Coexisting Conditions

The coexisting conditions examined varied across the included studies. The pooled analysis showed no statistical correlation between coexisting conditions and AR (**Supplementary Fig. 3**). The coexisting conditions evaluated in this meta-analysis were coronary heart disease (**Supplementary Fig. 3A**; OR = 1.03; 95% CI = 0.88 to 1.21; I^2^ = 0%; *p* = 0.91), hypertension (**Supplementary Fig. 3B**; OR = 1.04; 95% CI = 0.99 to 1.10; I^2^ = 35%; *p* = 0.17), previous myocardial infarction (**Supplementary Fig. 3C**; OR = 0.94; 95% CI = 0.67 to 1.33; I^2^ = 0%; *p* = 0.87), and previous stroke (**Supplementary Fig. 3D**; OR = 0.92; 95% CI = 0.60 to 1.40; I^2^ = 0%; *p* = 0.57).

#### 3.3.4 Correlation between AR and Laboratory Results

**Fig. 3.**
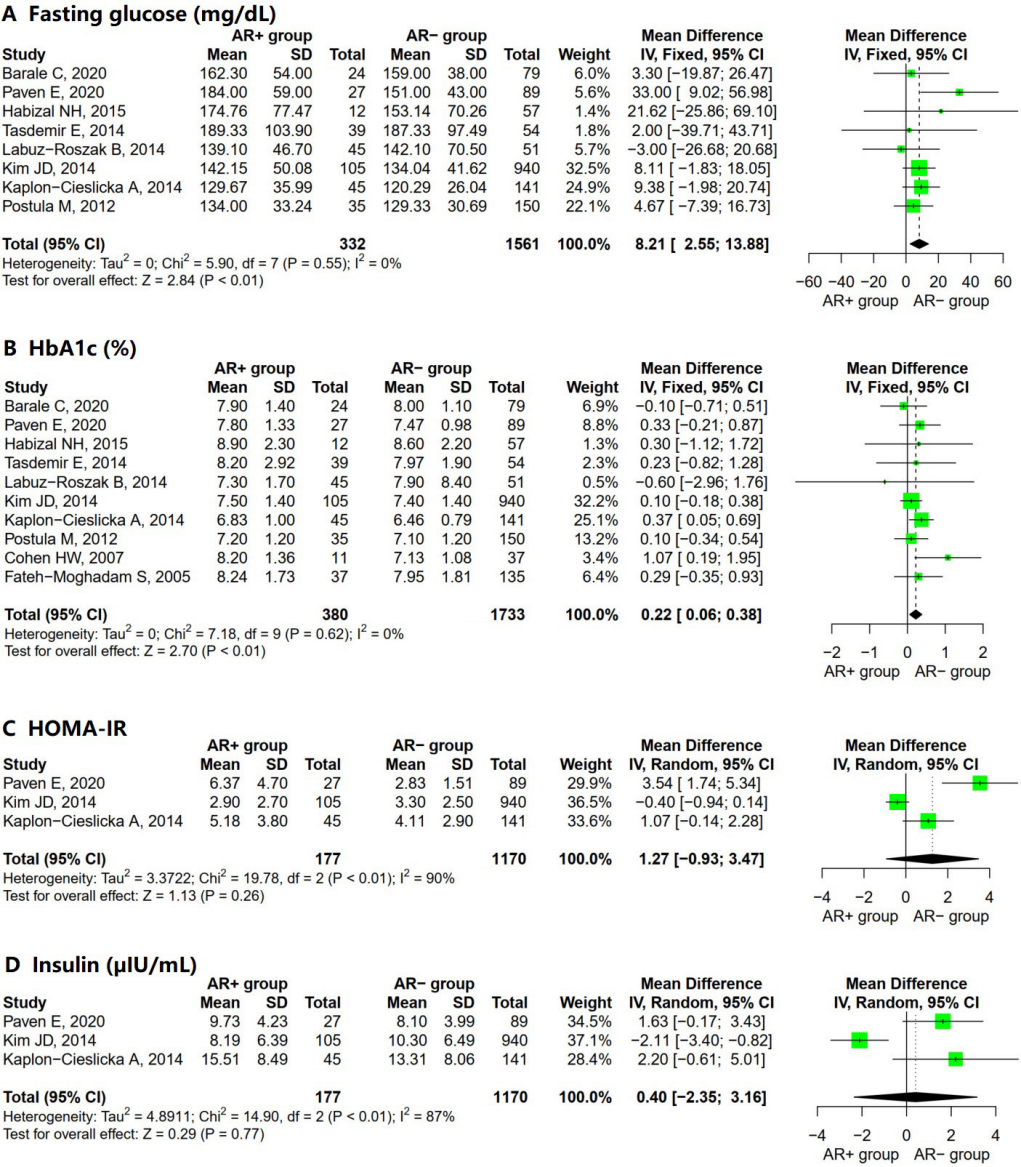
**Forest plot for AR+ vs. AR– regarding (A) fasting glucose, (B) HbA1c, (C) HOMA-IR, and (D) insulin**. Abbreviations: SD, standard deviation; IV, inverse variance method; CI, confidence interval; AR, aspirin resistance; HbA1c, glycated hemoglobin; HOMA-IR, Homeostasis Model Assessment-Insulin Resistance.

**Fig. 4.**
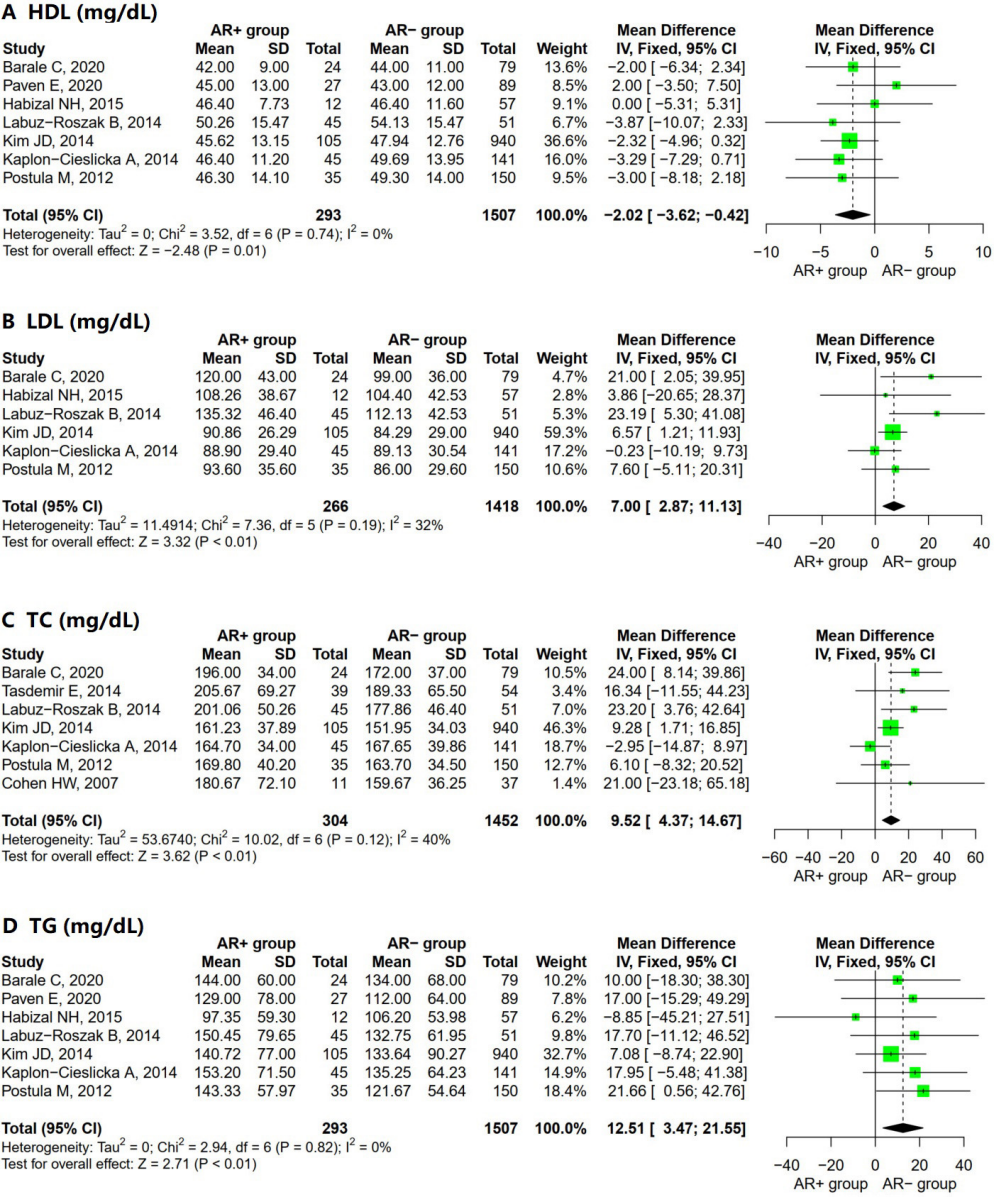
**Forest plot for AR+ vs. AR– regarding (A) HDL, (B) LDL, (C) TC, and (D) TG**. Abbreviations: HDL, high-density lipoprotein; LDL, low-density lipoprotein; TC, total cholesterol; TGs, triglycerides; SD, standard deviation; CI, confidence interval; IV, inverse variance method; AR, aspirin resistance.

The combined laboratory findings of patients with T2DM were organized into three subcategories: diabetic parameters (Fig. 3), lipid control parameters (Fig. 4), and other laboratory parameters (**Supplementary Fig. 4**). In the analysis of diabetic parameters, significative differences between the AR– and the AR+ group were found regarding fasting glucose level (Fig. 3A; MD = 8.21; 95% CI = 2.55 to 13.88; I^2^ = 0%; *p* = 0.55) and glycated hemoglobin (HbA1c) (Fig. 3B; MD = 0.22; 95% CI = 0.06 to 0.38; I^2^ = 0%; *p* = 0.62); the two parameters with no significant differences between the two groups were HOMA-IR index (Fig. 3C; MD = 1.27; 95% CI = –0.93 to 3.47; I^2^ = 90%; *p* < 0.01) and serum insulin level (Fig. 3D; MD = 0.40; 95% CI = –2.35 to 3.16; I^2^ = 87%; *p* < 0.01).

Statistically significant differences were observed in all pooled parameters analyzed in the lipid control parameters (Fig. 4). The AR+ group had a lower high-density lipoprotein (HDL) level (Fig. 4A; MD = –2.02; 95% CI = –3.62 to –0.42; I^2^ = 0%; *p* = 0.74) and a higher level of low-density lipoprotein (LDL) (Fig. 4B; MD = 7.00; 95% CI = 2.87 to 11.13; I^2^ = 32%; *p* = 0.19), total cholesterol (TC) (Fig. 4C; MD = 9.52; 95% CI = 4.37 to 14.67; I^2^ = 40%; *p* = 0.12), and triglycerides (TGs) (Fig. 4D; MD = 12.51; 95% CI = 3.47 to 21.55; I^2^ = 0%; *p* = 0.82) than the AR– group. The heterogeneity was low for all included lipid control parameters.

In analyzing other laboratory parameters (**Supplementary Fig. 4**), no positive correlation was identified between AR and any examined parameters. The measurements within this group were serum creatinine level (**Supplementary Fig. 4A**; MD = 1.95; 95% CI = –1.80 to 5.69; I^2^ = 9%; *p* = 0.35), eGFR (**Supplementary Fig. 4B**; MD = –0.14; 95% CI = –3.33 to 3.05; I^2^ = 0%; *p* = 0.66), hemoglobin level (**Supplementary Fig. 4C**; MD = 0.21; 95% CI = –0.01 to 0.43; I^2^ = 15%; *p* = 0.32), mean platelet volume (**Supplementary Fig. 4D**; MD = 0.14; 95% CI = –0.05 to 0.33; I^2^ = 25%; *p* = 0.25), and platelet count (**Supplementary Fig. 4E**; MD = 1.66; 95% CI = –11.62 to 14.95; I^2^ = 51%; *p* = 0.07).

### 3.4 Heterogeneity Assessment

The results of I^2^ and Cochran’s Q test from the meta-analysis (Figs. 2,3,4) revealed low heterogeneity among studies for all variables correlated with AR. However, further assessment is warranted to ascertain the stability and reliability of the aggregated outcomes due to inconsistency in the included studies (detailed in the Discussion section).

#### 3.4.1 Galbraith Test

**Fig. 5.**
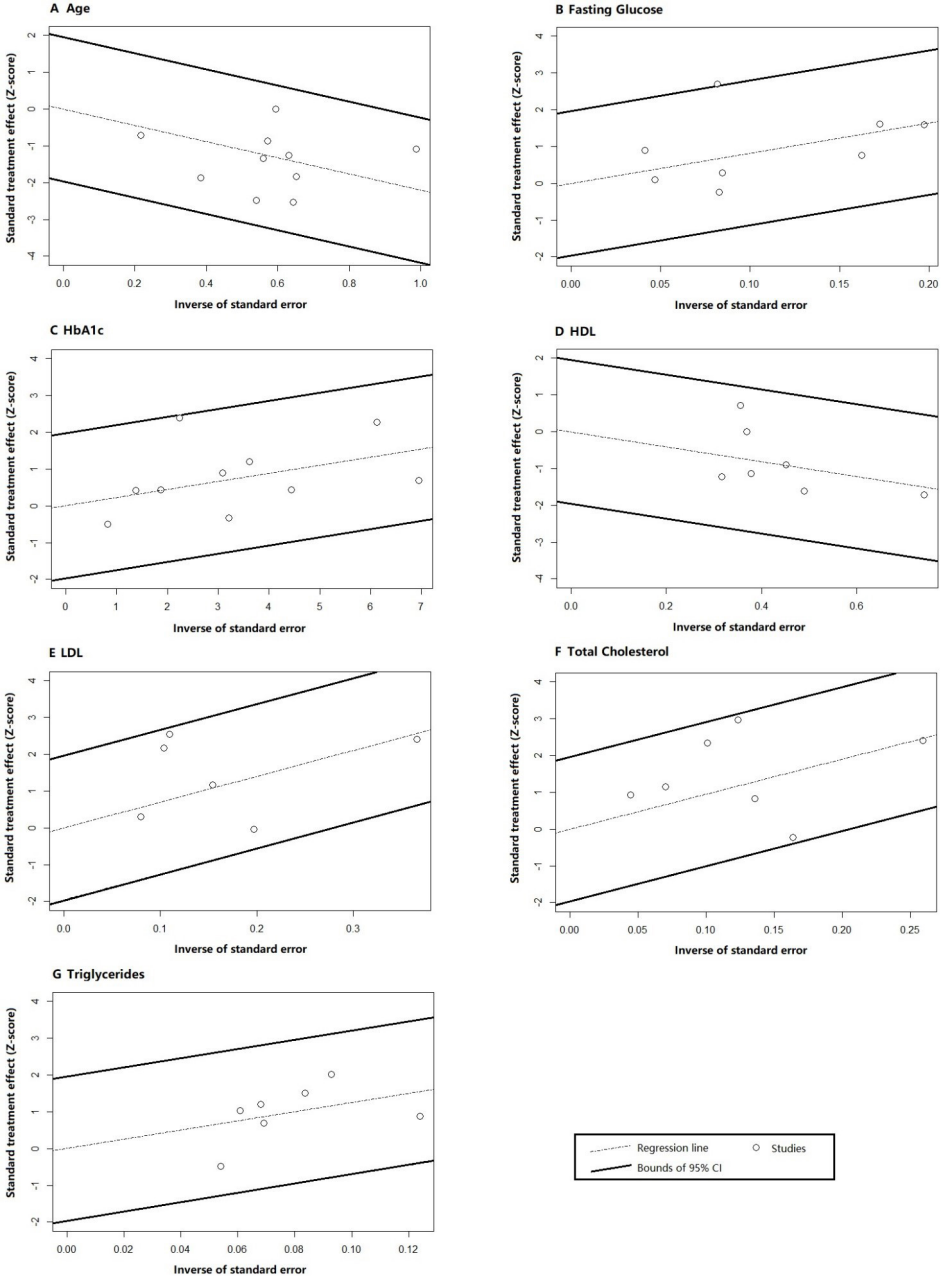
**Galbraith test results for (A) age, (B) fasting glucose, (C) HbA1c, (D) HDL, (E) LDL, (F) total cholesterol, and (G) triglycerides**. Abbreviations: CI, confidence interval; HDL, high-density lipoprotein; LDL, low-density lipoprotein; HbA1c, glycated hemoglobin.

Galbraith tests were performed to evaluate the existence of outliers and inconsistency among studies (Fig. 5). The plots illustrated that all individual results were within the 95% CI except for one study (Paven E, 2020 [27]) on fasting glucose. Since the deviation from the expected range was not significant, no obvious outlier or source of heterogeneity was identified.

#### 3.4.2 Sensitivity Analysis

The sensitivity analysis was designed to explore whether diversified AR detection methods caused heterogeneity among studies. According to the adopted platelet function testing approach, combined results excluding certain reports were sequentially generated and compared with originally synthesized data, as shown in Table 4. The sensitivity analysis suggested that the meta-analysis results were relatively stable. Only 2 out of 42 (4.7%) test scenarios exhibited a transformation from significant to non-significant differences between the AR+ and AR– groups (*p* > 0.05). Specifically, the two test cases were HbA1c omitting STI (MD = 0.17 (–0.01, 0.36), *p* = 0.07) and HDL omitting VNS (MD = –1.65 (–3.83, 0.53), *p* = 0.14). The impact of these two assays (STI and VNS) on heterogeneity should be further investigated.

**Table 4.** **Sensitivity analysis results of potential AR predictors**.

Parameter	Group	Number of included studies	Meta-analysis result		
			MD (95% CI)	*p*-value	I^2^ %
Age	Baseline	10	–2.21 (–3.23, –1.19)	<0.01	0
	Omitting PFA	6	–1.83 (–3.00, –0.66)	<0.01	0
	Omitting LTA	9	–2.44 (–3.51, –1.37)	<0.01	0
	Omitting TEG	9	–2.19 (–3.26, –1.12)	<0.01	0
	Omitting MPA	9	–2.13 (–3.21, –1.05)	<0.01	0
	Omitting VNS	8	–2.75 (–4.02, –1.49)	<0.01	0
	Omitting STI	9	–1.99 (–3.07, –0.91)	<0.01	0
Fasting glucose	Baseline	8	8.21 (2.55, 13.88)	<0.01	0
	Omitting PFA	6	8.65 (2.75, 14.56)	<0.01	11
	Omitting LTA	7	6.75 (0.91, 12.58)	0.02	0
	Omitting TEG	7	8.02 (2.31, 13.72)	<0.01	0
	Omitting MPA	7	8.89 (3.06, 14.73)	<0.01	0
	Omitting VNS	6	10.01 (1.6, 18.41)	0.02	7
	Omitting STI	7	7.82 (1.29, 14.36)	0.02	0
HbA1c	Baseline	10	0.22 (0.06, 0.38)	<0.01	0
	Omitting PFA	6	0.21 (0.03, 0.39)	0.02	0
	Omitting LTA	9	0.21 (0.04, 0.38)	0.01	0
	Omitting TEG	9	0.22 (0.06, 0.38)	<0.01	0
	Omitting MPA	9	0.22 (0.06, 0.39)	<0.01	0
	Omitting VNS	8	0.32 (0.1, 0.54)	<0.01	0
	Omitting STI	9	0.17 (–0.01, 0.36)	0.07	0
HDL	Baseline	7	–2.02 (–3.62, –0.42)	0.01	0
	Omitting PFA	6	–2.03 (–3.75, –0.31)	0.02	0
	Omitting LTA	6	–2.4 (–4.07, –0.72)	<0.01	0
	Omitting TEG	6	–2.23 (–3.9, –0.55)	<0.01	0
	Omitting MPA	6	–1.89 (–3.55, –0.23)	0.03	0
	Omitting VNS	5	–1.65 (–3.83, 0.53)	0.14	0
	Omitting STI	6	–1.78 (–3.53, –0.06)	0.03	0
LDL	Baseline	6	7.00 (2.87, 11.13)	<0.01	32
	Omitting PFA	5	6.31 (2.08, 10.54)	<0.01	23
	Omitting LTA	6	7.00 (2.87, 11.13)	<0.01	32
	Omitting TEG	5	7.10 (2.91, 11.28)	<0.01	45
	Omitting MPA	5	6.09 (1.85, 10.34)	<0.01	1
	Omitting VNS	4	7.65 (0.12, 15.18)	0.03	59
	Omitting STI	5	(3.97, 13.04)	<0.01	19
TC	Baseline	7	9.52 (4.37, 14.67)	<0.01	40
	Omitting PFA	4	7.26 (1.67, 12.85)	0.01	47
	Omitting LTA	7	9.52 (4.37, 14.67)	<0.01	40
	Omitting TEG	7	9.52 (4.37, 14.67)	<0.01	40
	Omitting MPA	6	8.49 (3.15, 13.82)	<0.01	37
	Omitting VNS	5	10.85 (2.81, 18.89)	<0.01	59
	Omitting STI	6	12.38 (6.67, 18.09)	<0.01	0
TGs	Baseline	7	12.51 (3.47, 21.55)	<0.01	0
	Omitting PFA	6	12.80 (3.26, 22.34)	<0.01	0
	Omitting LTA	6	12.13 (2.71, 21.55)	0.01	0
	Omitting TEG	6	13.92 (4.59, 23.25)	<0.01	0
	Omitting MPA	6	11.95 (2.42, 21.47)	0.01	0
	Omitting VNS	5	12.71 (1.21, 25.63)	0.02	0
	Omitting STI	6	11.56 (1.76, 21.36)	0.02	0

Abbreviations: HDL, high-density lipoprotein; LDL, low-density lipoprotein; TC, total cholesterol; TGs, triglycerides; PFA, platelet function analyzer; LTA, light transmission aggregometry; TEG, thromboelastography; MPA, multiplate analyzer; VNS, VerifyNow system; STI, serum TXB2 immunoassay; MD, mean difference; CI, confidence interval; HbA1c, glycated hemoglobin; AR, aspirin resistance.

#### 3.4.3 Subgroup Analysis

Seven studies [16, 17, 19, 27, 29, 30, 31] ensured consistency in aspirin dosage among participants, with a daily intake of either 75 mg or 100 mg (see Table 2). Patients were administered a varying dose of aspirin in the three remaining studies. The results of the subgroup analysis by aspirin dose (fixed/flexible) are shown in Table 5. The difference between AR+ and AR– groups was significant in all fixed aspirin dose subgroups. In contrast, in the flexible aspirin dose subgroups, the analysis of three laboratory parameters demonstrated no significant difference between AR+ and AR– patients (fasting glucose: MD = 1.90 (–19.28, 23.09), *p* = 0.86; HDL: MD = –1.64 (–5.67, 2.40), *p* = 0.43; TG: MD = 7.46 (–15.13, 30.04), *p* = 0.52). While variations in aspirin dosage may introduce heterogeneity into the study, further validation is essential before determining definitive conclusions.

**Table 5.** **Subgroup analyses of AR-related parameters**.

Parameter	ASA dose subgroup	Number of included studies	Meta-analysis result		
			MD (95% CI)	*p*-value	I^2^ %
Age	Fixed	7	–2.08 (–3.23, –0.93)	<0.01	14
	Flexible	3	–2.67 (–4.89, –0.46)	0.02	0
Fasting glucose	Fixed	6	8.70 (2.82, 14.58)	<0.01	0
	Flexible	2	1.90 (–19.28, 23.09)	0.86	0
HbA1c	Fixed	7	0.19 (0.03, 0.36)	0.01	0
	Flexible	3	0.73 (0.01, 1.44)	0.05	7
HDL	Fixed	5	–2.10 (–3.84, –0.35)	0.01	0
	Flexible	2	–1.64 (–5.67, 2.40)	0.43	0
LDL	Fixed	4	6.16 (1.85, 10.47)	<0.01	25
	Flexible	2	16.47 (2.02, 30.92)	0.03	36
TC	Fixed	5	8.30 (2.92, 13.68)	<0.01	38
	Flexible	2	22.84 (5.05, 40.63)	0.01	0
TGs	Fixed	5	13.48 (3.61, 23.34)	<0.01	0
	Flexible	2	7.46 (–15.13, 30.04)	0.52	21

Abbreviations: HDL, high-density lipoprotein; LDL, low-density lipoprotein; TC, total cholesterol; TGs, triglycerides; ASA, acetylsalicylic acid; MD, mean difference; CI, confidence interval; AR, aspirin resistance; HbA1c, glycated hemoglobin.

#### 3.4.4 Meta-Regression 

Meta-regression analysis was adopted to evaluate the heterogeneity attributed to aspirin dose (fixed versus flexible dose) and AR detection techniques (STI and VNS) on the observed heterogeneity. The results suggested that aspirin dose and the adoption of STI and VNS were not significant contributors to heterogeneity in the findings of the present study; detailed results are presented in Table 6.

**Table 6.** **Results of univariate meta-regression analysis**.

Covariate	Outcome	Standard error	Coefficient (95% CI)	*p*-values
Aspirin dose	Age	1.28	–0.59 (–3.09, 1.91)	0.65
	Fasting glucose	11.22	–6.80 (–28.80, 15.19)	0.54
	HbA1c	0.37	0.53 (–0.20, 1.26)	0.15
	HDL	2.24	0.46 (–3.93, 4.85)	0.84
	LDL	7.69	10.31 (–4.77, 25.39)	0.18
	TC	11.39	13.89 (–8.44, 36.22)	0.22
	TGs	12.58	–6.02 (–30.67, 18.63)	0.63
AR detection: STI	Age	1.65	1.95 (–1.28, 5.19)	0.24
	Fasting glucose	6.69	–1.56 (–14.67, 11.55)	0.82
	HbA1c	0.19	–0.20 (–0.57, 0.17)	0.29
	HDL	2.22	1.51 (–2.86, 5.87)	0.50
	LDL	6.94	10.08 (–3.53, 23.69)	0.15
	TC	8.15	16.43 (–4.46, 32.41)	0.14
	TGs	12.96	–6.39 (–31.78, 19.00)	0.62
AR detection: VNS	Age	1.09	–1.55 (–3.69, 0.58)	0.15
	Fasting glucose	5.81	3.29 (–8.01, 14.67)	0.57
	HbA1c	0.16	0.22 (–0.10, 0.54)	0.18
	HDL	1.64	0.81 (–2.40, 4.02)	0.62
	LDL	7.96	3.29 (–12.30, 18.88)	0.68
	TC	9.71	5.88 (–13.15, 24.91)	0.54
	TGs	9.23	0.38 (–17.71, 18.47)	0.97

Abbreviations: STI, serum TXB2 immunoassay; VNS, VerifyNow system; HDL, high-density lipoprotein; LDL, low-density lipoprotein; TC, total cholesterol; TGs, triglycerides; CI, confidence interval; AR, aspirin resistance; HbA1c, glycated hemoglobin.

## 4. Discussion

Our systematic review and meta-analysis focused on comparing the clinical characteristics of AR versus non-AR among diabetic patients receiving aspirin treatment. The main findings can be summarized as follows: (1) AR is associated with younger patients, whereas there were no significant differences in gender distribution, BMI, and smoking status between the two groups. (2) Non-AR patients exhibit similar profiles of coexisting conditions and concurrent medications compared to AR patients. (3) Regarding laboratory results examined in this meta-analysis, all lipid control parameters (HDL, LDL, TG, and TC levels) and two diabetic parameters (fasting glucose and HbA1c) demonstrated significant correlations with AR.

Aspirin resistance is a prevalent clinical phenomenon and is empirically defined as a condition where the conventional dose of aspirin fails to exhibit consistent antiplatelet effects [32]. This ambiguous definition leads to inconsistencies in various aspects of research concerning AR. Firstly, there is a lack of standardized aspirin dosage; a daily dose of aspirin, recommended by the American Society for Vascular Surgery, is between 75 and 325 mg as a secondary prevention strategy against adverse cardiovascular complications [33]. In this meta-analysis, patients from the included studies were administered varying doses of aspirin, ranging from 75 to 325 mg daily. Secondly, AR is currently verified by various platelet function tests [34]. Certain assays have been adopted in clinical practice based on considerations such as sensitivity in test results, availability of resources, and simplicity of use [34]. Six AR assays were identified in the included papers. Several heterogeneity tests were performed to assess the potential influence of heterogeneity in combined results caused by aspirin doses and AR detections. The I^2^ test, Cochran’s Q test, and Galbraith test all suggested that the heterogeneity of the included studies was generally low. After sensitivity analysis, two AR tests (STI and VNS) were doubtful, necessitating further investigation. Subgroup analysis by aspirin dose showed that the significant differences in three laboratory parameters (TG, HDL, and fasting glucose) between AR and no-AR groups became non-significant in patients receiving various doses of aspirin (between 75 to 325 mg). Conversely, the meta-regression analysis results concluded that aspirin dose and AR assays were not the primary source of heterogeneity. Roller *et al*. [35] investigated the impact of aspirin dose on AR and reported that increasing the aspirin dose did not convert aspirin non-responders into responders. Aspirin non-responders were defined as possessing a collagen and epinephrine closure time (CEPI-CT) exceeding 165 s. Their study confirmed AR individuals using the PFA-100 value among participants treated with 100 mg ASA daily for 7 days. Five identified ASA non-responders were re-examined after taking 300 mg ASA daily for three weeks, and none exhibited a transition to aspirin responders. Clinical evidence also suggested that higher doses do not enhance the cardioprotective effects of aspirin [36]. On the other hand, aspirin formulations (plain aspirin or enteric-coated) also had no significant impact on aspirin responsiveness [37, 38]. In summary, our study suggests minimal heterogeneity attributed to aspirin doses. While each laboratory assay for AR has inherent limitations [34], our subgroup analysis and meta-regression results demonstrated that AR assays are not a significant source of heterogeneity. This conclusion is consistent with findings from other meta-analysis reports [39, 40], which combined results focusing on clinical outcomes among AR patients. The argument is that since each individual assay reveals a certain level of rationality and sensitivity, any genuine clinical effects on screened AR individuals should also be observable [39].

Aspirin achieves its primary antithrombotic effect by acetylating the serine-529 residue of COX-1 irreversibly [41]. COX-1 induces the conversion of arachidonic acid into thromboxane A2 (TXA2, which is a potent platelet activator that binds to the TXA2 receptor (TP) expressed on platelet membranes, thereby initiating the TP-mediated platelet aggregation pathway [41, 42]. Briefly, aspirin suppresses platelet activation by blocking the synthesis of COX-1-dependent TXA2. While the inhibition of COX-1 by aspirin is rapid, irreversible, saturable at low doses, and sustained throughout the lifespan of a platelet (7–10 days) [43], the prevalence of AR in patients with T2DM could be up to 60% depending on the measurements used [44]. In our pooled analysis, AR was mainly associated with increased insulin resistance and poor lipid control indicators. The predictors found in this study play crucial roles in the mechanisms of AR in diabetes. Firstly, hyperglycemia and hypercholesterolemia are thought to cause endothelial dysfunction through elevated oxidative stress and impaired nitric oxide (NO) biosynthesis and transportation [12, 45]. Endothelial dysfunction mediates platelet activation and adhesion to endothelial cells, causing rapid platelet generation and heavy platelet consumption [12]. The resulting enhanced platelet turnover leads to the production of immature platelets, which are rich in mRNA and could generate unacetylated COX-1 and COX-2 [11]. Given the short half-life of aspirin, the newly formed COX-1 may not be adequately inhibited. Consequently, activating the COX-1-dependent TXA2 pathway leads to the aspirin treatment failing. Subsequently, the glycated platelet membrane proteins are structurally altered and become less accessible for acetylation, making aspirin less effective [11]. Secondly, COX-2, the second isoform of COX, is typically found in less than 10% of resting platelets; however, its expression is upregulated in inflammatory conditions by accelerated platelet turnover. Notably, COX-2 is not sensitive to low-dose aspirin and induces the production of TXA2 in a COX-1-independent manner [46]. Platelets can also be activated by another COX-1-independent pathway, which entails the oxidation of arachidonic acid and subsequent generation of isoprostanes—aspirin-insensitive agonists that bind the TXA2 receptor and activate platelets [41]. Interestingly, platelet aggregation still occurs in the presence of isoprostanes, even though TXA2 levels are significantly decreased by aspirin [14, 41]. In conclusion, hyperglycemia and dyslipidemia play significant roles in both the COX-1-dependent and COX-1-independent platelet activation pathways.

In addition to the previously discussed results, further observations from the current study are briefly outlined. An additional association was observed between AR and younger patients. This finding could be elucidated from a pharmacokinetic standpoint, as aspirin esterase activity diminishes in older individuals, particularly those with heightened inflammatory conditions [16, 47]. Given that most participants in this study were administered a relatively low aspirin dose (no more than 100 mg per day), the presence of AR in younger patients could potentially be linked to a heightened rate of aspirin hydrolysis. Intriguingly, despite insulin levels and HOMA-IR being considered reliable indicators of insulin resistance, they did not show an association with AR in our analysis. This discrepancy could be partly attributed to the limited number of studies (only three) that investigated insulin levels and HOMA-IR, leading to notable heterogeneity in the pooled results (*p* < 0.01) and thereby casting doubt on the findings. Furthermore, the absence of a robust correlation between AR and coexisting vascular diseases may be rationalized by the shared underlying mechanisms of AR in both diabetes and vascular disorders [48]. Therefore, it is plausible that prior vascular events may not exert a significant impact on the development of AR in diabetic patients, especially considering their existing chronic inflammatory state and elevated oxidative stress levels attributed to inadequate glucose management and hyperlipidemia. Conversely, our findings indicate that BMI, although identified as a determinant of AR in certain studies [18, 29], may not be reliable as a marker of dyslipidemia and hyperglycemia.

Our study has several limitations. First, regarding the AR study, most available randomized clinical trials (RCTs) and meta-analyses focused on the efficacy and safety of aspirin treatment as a first or second prevention strategy for vascular events. Notably, no RCTs specifically investigating the clinical predictors of AR in diabetic patients were identified. As a result, all publications included in this meta-analysis were observational studies shadowed by limited sample sizes and methodological issues, such as selection bias and confounding and controversial causal claims. Although the selection bias attributed to the non-randomized selection of intervention and control groups is unavoidable, all included studies adopted logistic regressions to reduce confounding. Notably, propensity score matching was not conducted in any of the selected studies, and sample size constraints might be a major concern. To evaluate the publication bias in our analysis, a funnel plot and Egger’s test were used, and the test results suggested that the conclusions of our meta-analysis were not skewed by publication bias. Second, possible sources of heterogeneity might be derived from variations in AR laboratory detection, ASA dosage, duration of treatment, and clinical characteristics of enrolled patients. Thus, Cochran’s Q test, I^2^ test, and Galbraith plot were conducted to assess the overall heterogeneity in our findings, while subgroup tests, sensitivity analyses, and univariable regression were also used to examine the distinctive differentials. Despite substantial variations, our study revealed minimal evidence of significant heterogeneity, underscoring the clinical clarity and specificity of our results. Finally, the factors and underlying mechanisms discussed above may not be sufficient to understand AR. Indeed, a wide diversity of aspirin pharmacodynamics and pharmacokinetics is equally important, if not more critical, in regulating aspirin metabolism, which includes processes such as absorption, bioavailability, and the excretion of this antiplatelet agent [15]. This makes us believe that AR is personalized and has multiple causes. To delve deeper into this issue, population-based longitudinal studies are urged to resolve meaningful questions. These questions include whether AR could be categorized as genetically determined (permanent) and acquired (temporary), elucidating the inheritance patterns of AR, investigating the duration of transient AR, and exploring the potential reversibility of AR status under specific conditions (health status and lifestyle). We conjecture that enhanced glucose and lipids levels may contribute to AR, although further evidence is required before determining any conclusions.

Aspirin is affordable, widely accessible, and a commonly used antiplatelet drug. Moreover, aspirin therapy is endorsed by the ADA as a secondary prevention measure for T2DM patients with a history of atherosclerotic cardiovascular disease [4]. This study and follow-up research could potentially positively impact clinical practices. While prescribing aspirin to T2DM patients for continuous therapy against adverse cardiovascular outcomes, we suggest that patients should be fully informed about the risk of poor lipids and glucose control. Since the risks extend beyond diabetes-related syndrome, the weakened antiplatelet capacity of aspirin makes patients vulnerable to unfavorable cardiovascular complications. This risk might be remarkably reduced by following the best practices in lipid and glucose control and regularly taking glucose and lipid profile blood tests, even though the prescription of aspirin therapy remains unchanged. AR assays may be less effective in accessing a dynamic internal environment with fluctuating glucose levels, lipids, and numerous macromolecules in the long term. Fortunately, advancements in understanding and managing these intricate dynamics offer opportunities for regulating and leveraging their potential benefits.

## 5. Conclusions

The current meta-analysis demonstrates that glucose levels and dyslipidemia markers effectively predict aspirin resistance in individuals diagnosed with T2DM. Further studies are needed to deepen this understanding, and the findings of our analysis and subsequent research may positively impact aspirin therapy among diabetic patients.

## Data Availability

The datasets used in our study are available from the corresponding author on reasonable request.

## Abbreviations

ACE, angiotensin-converting enzyme; ADA, American Diabetes Association; AR, aspirin resistance; ASA, acetylsalicylic acid; BMI, body mass index; CEPI-CT, collagen and epinephrine closure time; CI, confidence interval; COX-1, cyclooxygenase-1; COX-2, cyclooxygenase-2; CVD, cardiovascular disease; eGFR, estimated glomerular filtration rate; GRADE, Grading of Recommendations, Assessment, Development, and Evaluations; HbA1c, glycated hemoglobin; HDL, high-density lipoprotein; HOMA-IR, Homeostasis Model Assessment-Insulin Resistance; HPR, high platelet reactivity; IQR, interquartile range; IV, inverse variance method; LDL, low-density lipoprotein; LTA, light transmission aggregometry; MD, mean difference; MPV, mean platelet volume; MI, myocardial infarction; MH, Mantel–Haenszel model; MPA, multiplate analyzer; NO, nitric oxide; NOS, Newcastle–Ottawa scale; PFA, platelet function analyzer; PLT, platelet count; RCTs, randomized clinical trials; STI, serum thromboxane B2 immunoassay; TC, total cholesterol; TEG, thromboelastography; TGs, triglycerides; TI, TXB2 immunoassay; TP, TXA2 receptor; TXA2, thromboxane A2; TXB2, thromboxane B2; T2DM, type 2 diabetes mellitus; VNS, VerifyNow system.

## Supplementary Material

Supplementary material associated with this article can be found, in the online version, at https://doi.org/10.31083/RCM26009.
