# Supplementary material for: Clinical Predictors of Aspirin Resistance in Patients with Type 2 Diabetes: A Systematic Review and Meta-Analysis
Source: Rev Cardiovasc Med. 2025 Jan 20;26(1):26009. doi: 10.31083/RCM26009 (PMC11759956; doi:10.31083/RCM26009)
Supplement: Supplementary file 1 [file 2153-8174-26-1-26009-s1.zip › Supplementary Material.docx]

**Supplementary Table 1. Newcastle-Ottawa Scale (NOS) score.**

| Author,Year | Selection(0-4) | | | | Comparability  (0-2) | Exposure  (0-3) | | | Total |
| --- | --- | --- | --- | --- | --- | --- | --- | --- | --- |
|  | Adequate  definition | Representativeness | Selection of controls | Definition of Controls |  | Ascertainment of exposure | Same method | Non-Response rate |  |
| Barale, 2020 | 1 | 1 | 0 | 1 | 2 | 1 | 1 | 0 | 7 |
| Paven, 2020 | 1 | 0 | 0 | 1 | 2 | 1 | 1 | 0 | 6 |
| Habizal, 2015 | 1 | 0 | 0 | 1 | 2 | 1 | 1 | 0 | 6 |
| Tasdemir, 2014 | 1 | 0 | 0 | 1 | 2 | 1 | 1 | 0 | 6 |
| Łabuz-Roszak, 2014 | 1 | 1 | 0 | 1 | 1 | 1 | 1 | 0 | 6 |
| Kim, 2014 | 1 | 0 | 0 | 1 | 2 | 1 | 1 | 0 | 6 |
| Kaplon-Cieslicka, 2014 | 1 | 1 | 0 | 1 | 2 | 1 | 1 | 0 | 7 |
| Postula, 2012 | 1 | 0 | 0 | 1 | 2 | 1 | 1 | 0 | 6 |
| Cohen, 2007 | 1 | 1 | 0 | 1 | 2 | 1 | 1 | 0 | 7 |
| Fateh-Moghadam, 2005 | 1 | 1 | 0 | 1 | 2 | 1 | 1 | 0 | 7 |

**Supplementary Table 2. Egger’s test of publication bias.**

| Parameters | P-value |
| --- | --- |
| 1 demographic characteristics |  |
| 1.1 Age (years) | 0.18 |
| 1.2 Female Gender, n (%) | 0.15 |
| 1.3 BMI (kg/m2) | 0.21 |
| 1.4 Current smoker, n (%) | 0.48 |
| 2 concurrent medications |  |
| 2.1 ACE inhibitors, n (%) | 0.39 |
| 2.2 Beta-blockers, n (%) | 0.97 |
| 2.3 Calcium channel blockers, n (%) | 0.09 |
| 2.4 Statins, n (%) | 0.25 |
| 3 Coexisting conditions |  |
| 3.1 Coronary heart disease, n (%) | 0.67 |
| 3.2 Hypertension, n (%) | 0.71 |
| 3.3 Previous MI, n (%) | 0.53 |
| 3.4 Previous stroke, n (%) | 0.89 |
| 4 Laboratory results |  |
| 4.1 diabetic parameters |  |
| 4.1.1 Fasting glycemia (mg/dL) | 0.68 |
| 4.1.2 HbA1c (%) | 0.76 |
| 4.1.3 HOMA-IR | 0.11 |
| 4.1.4 Insulin (µIU/mL) | 0.36 |
| 4.2 lipid control parameters |  |
| 4.2.1 HDL (mg/dL) | 0.55 |
| 4.2.2 LDL (mg/dL) | 0.37 |
| 4.2.3 TC (mg/dL) | 0.35 |
| 4.2.4 TG (mg/dL) | 0.9 |
| 4.3 Other laboratory parameters |  |
| 4.3.1 Creatinine (µmol/L) | 0.41 |
| 4.3.2 eGFR (mL/min/1.73m2) | 0.97 |
| 4.3.3 Haemoglobin (g/dL) | 0.74 |
| 4.3.4 Mean platelet volume (fL) | 0.08 |
| 4.3.5 Platelet count (1000/mm3) | 0.19 |


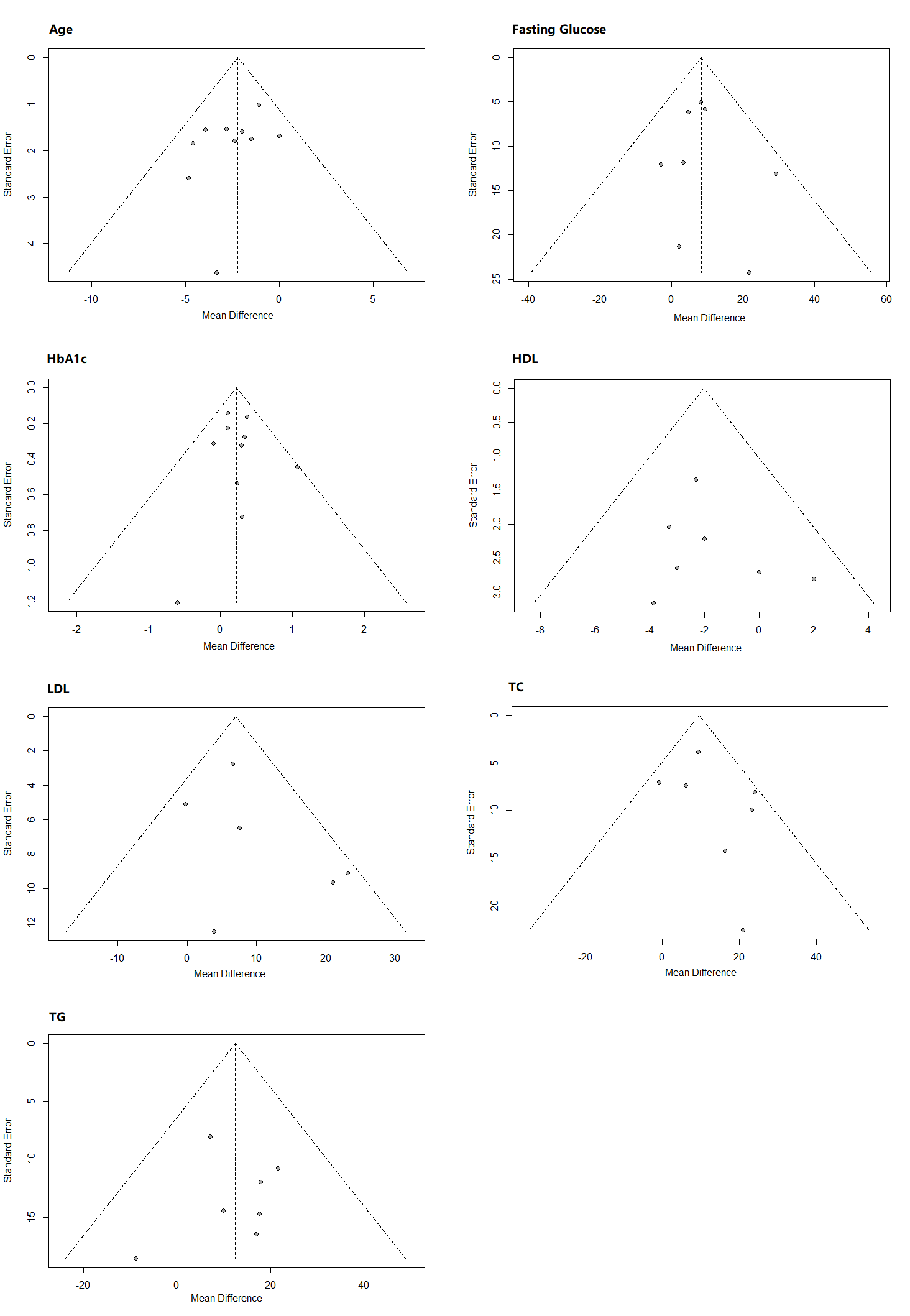


**Supplementary Fig. 1. Funnel plot for age, fasting glucose, HbA1c, HDL, LDL, TC, and TG.**


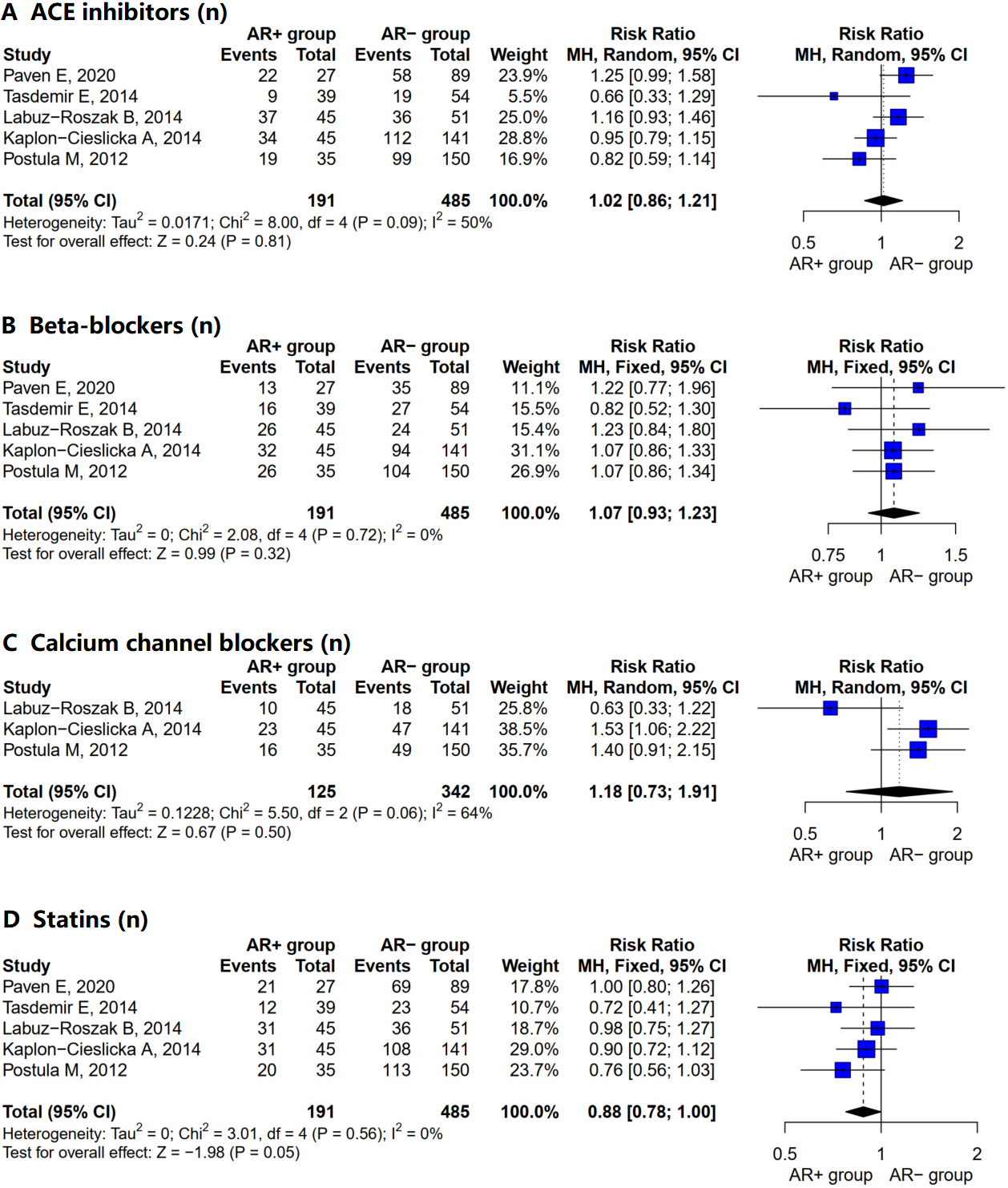


**Supplementary Fig. 2.** Forest plot for AR+ vs. AR- regarding **A** ACE inhibitors, **B** Beta-blockers, **C** Calcium channel blockers, **D** Statins.


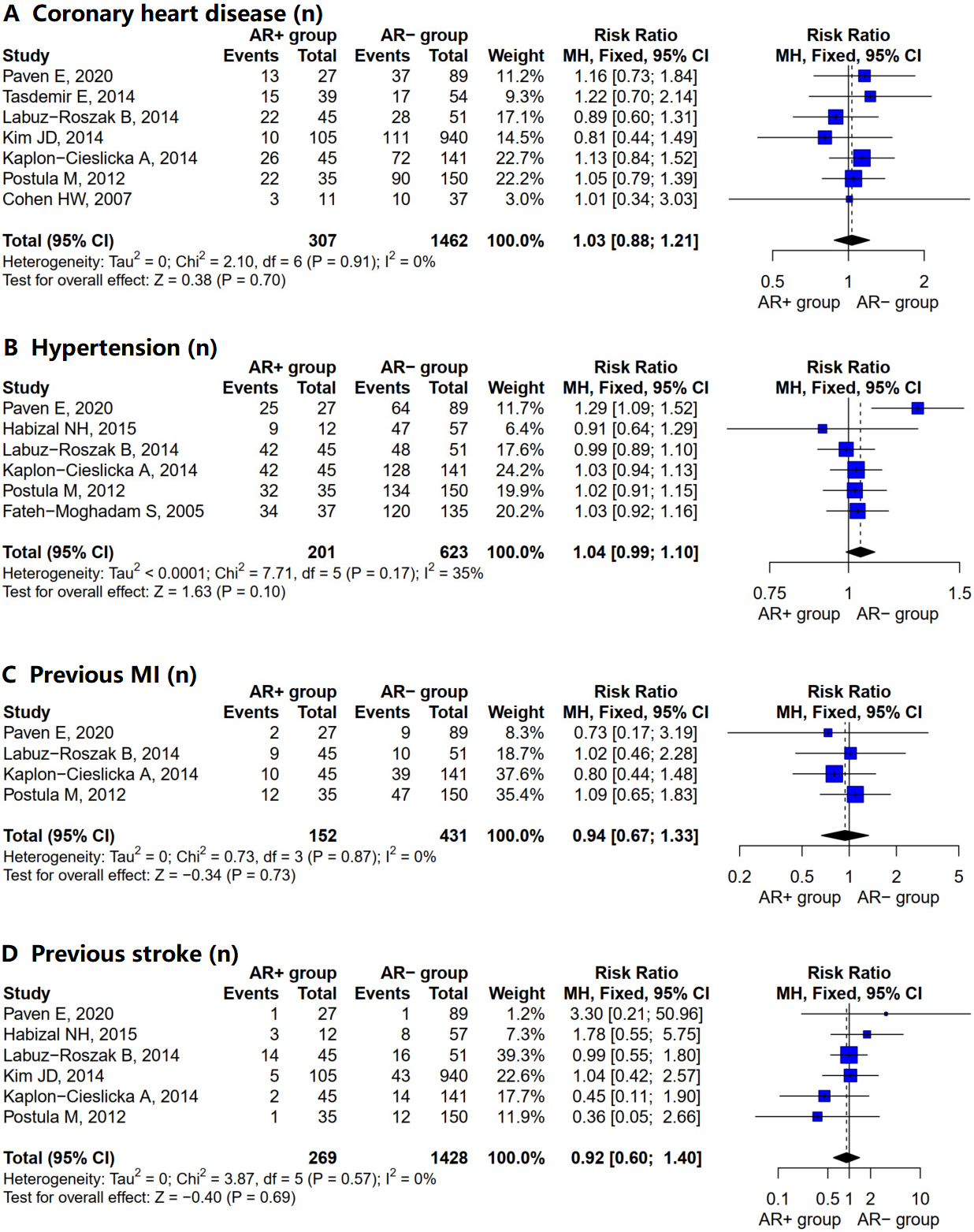


**Supplementary Fig. 3.** Forest plot for AR+ vs. AR- regarding **A** Coronary heart disease, **B** Hypertension, **C** Previous MI, **D** Previous stroke.


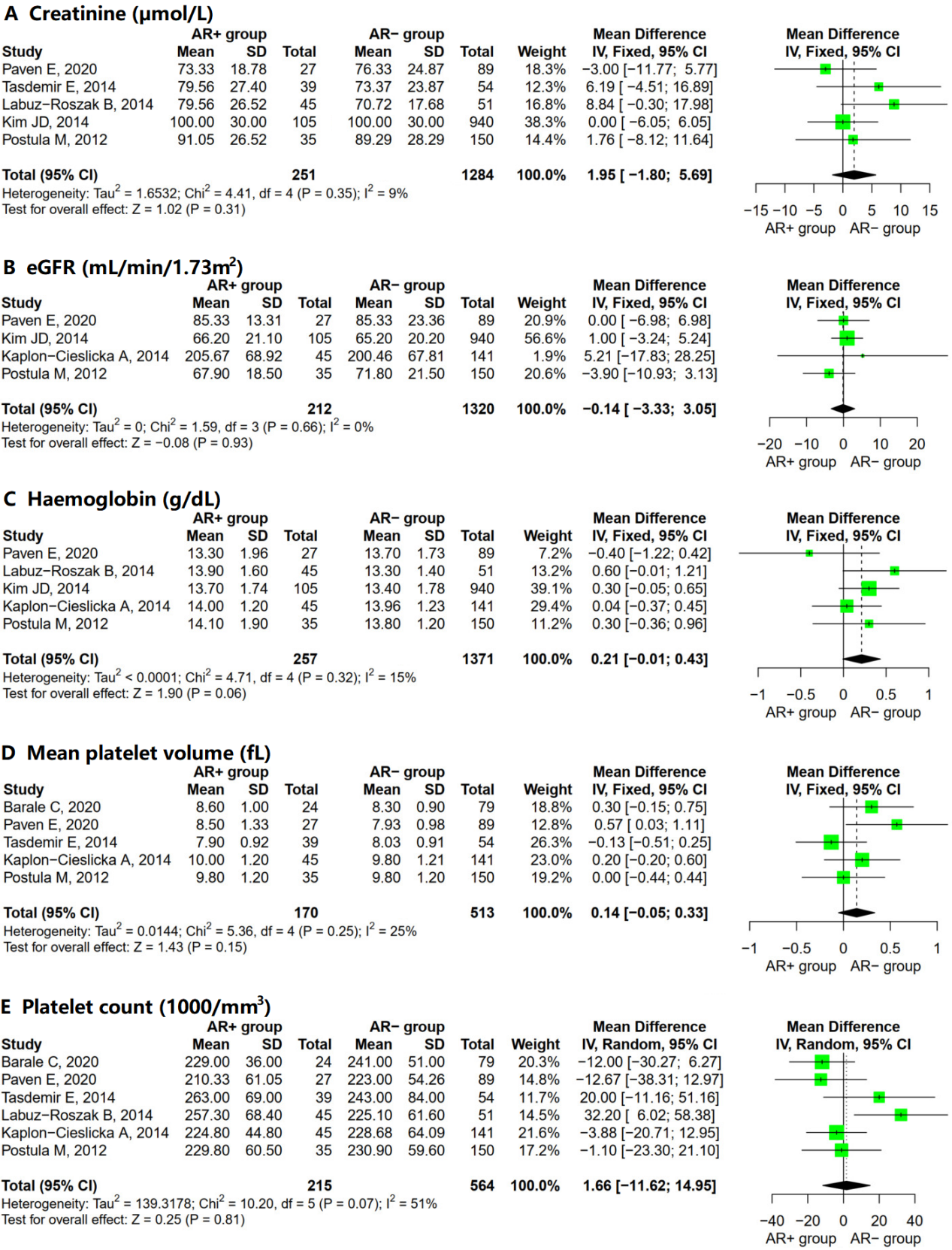


**Supplementary Fig. 4.** Forest plot for AR+ vs. AR- regarding **A** Creatinine, **B** eGFR, **C** Haemoglobin, **D** Mean platelet volume, **E** Platelet count.
